# Cancer detection using whole-genome sequencing of cell free DNA

**DOI:** 10.18632/oncotarget.1183

**Published:** 2013-07-15

**Authors:** Rebecca J. Leary, Mark Sausen, Luis A. Diaz, Victor E. Velculescu

**Affiliations:** Ludwig Center for Cancer Genetics, Johns Hopkins Kimmel Cancer Center, Baltimore, MD, USA. Present address: Novartis Institutes for Biomedical Research, Cambridge, Massachusetts, USA; Ludwig Center for Cancer Genetics, Johns Hopkins Kimmel Cancer Center, Baltimore, MD, USA. Present address: Personal Genome Diagnostics, Baltimore, MD, USA; Ludwig Center for Cancer Genetics, Johns Hopkins Kimmel Cancer Center, Baltimore, MD, USA; Ludwig Center for Cancer Genetics, Johns Hopkins Kimmel Cancer Center, Baltimore, MD, USA

Biomarkers are molecules whose detection provide valuable information regarding human disease. They may herald the presence of a disease or provide information regarding the course of disease and response to treatment. Historically, cancer biomarkers have been based on aberrant protein expression in a subset of tumor types (e.g. CEA in colorectal cancer, PSA in prostate cancer, and CA19-9 in ovarian cancer). Although protein biomarkers have been useful in cancer over the past several decades, their correlation with disease could be improved by the development of more sensitive, specific, and widely applicable cancer biomarkers. We have recently developed a non-invasive approach using whole-genome sequencing to identify chromosomal alterations in the circulation of cancer patients [1]. Cell-free DNA analyses of the circulation have the potential to alter the management of cancer care, leading to earlier diagnoses, identification of disease recurrence, and monitoring responses to therapy.

Human cancer arises from the sequential accumulation of genetic mutations. Dozens of genetic changes may be observed in each tumor's genome and are comprised of small sequence alterations as well as structural alterations, including copy number changes and rearrangements. Over the past decade genome sequencing has elucidated complex cancer genome landscapes, with unique collections of somatic genetic alterations in each patient. The phenomena of chromosomal instability is thought to be near-universal in human cancers and responsible for the development of somatic structural alterations. Recent analyses of structural alterations in breast cancers have shown multiple alterations in every cancer genome evaluated [2]. Additionally, analyses of 81 colorectal and breast cancer cell lines and xenografts using SNP arrays showed at least five whole-chromosome or chromosome arm alterations and an average of 14 focal copy number changes per case [3]. Although the same genes may not be altered in patients with a particular tumor type, the universal phenomena of somatic genetic alterations may be exploited to develop biomarkers that are specific to each patient's tumor.

Structural alterations, including rearrangements and chromosome arm gains and losses, are especially attractive biomarkers as they are dramatic changes that readily distinguish tumor-derived DNA from normal DNA. When primary tumor material is available, patient-specific rearrangements may be identified with Personalized Analysis of Rearranged Ends (PARE) or related methods [4,5]. Using massively parallel sequencing, PARE identifies rearrangements in the tumor and allows development of personalized PCR biomarker analyses to detect patient-specific rearrangements in cell-free DNA present in the circulation. PARE may be used to monitor disease progression, determine response to therapy, and evaluate presence of residual disease after potentially curative surgery or other clinical interventions.

Given the potential applications of these approaches, we recently examined whether we could extend this method to identify structural alterations directly in patient plasma without prior knowledge of disease status or tumor genotype [1]. Cell-free DNA isolated from patient plasma was used to generate libraries for massively parallel sequencing. The sequencing data obtained from whole genome sequencing of cell-free DNA permitted detection of both rearrangements as well as larger chromosomal abnormalities such as gains or losses of whole chromosomes or chromosome arms. In this proof of principle study, we sequenced cell-free DNA from the plasma of seven colorectal and three breast cancer patients and successfully detected structural alterations in the plasma of all cases analyzed. When these same analyses were applied to plasma samples from 10 healthy individuals as well as 28 normal lymphocyte samples, no structural alterations were identified.

Several of the rearranged sequences detected in patient plasma were in regions associated with clinically actionable genes. As genomic rearrangements co-occur with copy number alterations, the detected rearrangements could be used to infer the presence of copy number changes in nearby genes. For example, rearrangements associated with the *ERBB2* oncogene were identified in the plasma of a colorectal cancer patient and shown to be the result of *ERBB2* amplification in the patient's tumor. These results indicate the potential application of this approach to identify clinically relevant tumor-specific genetic changes in the absence of available tumor tissue [1].

Although this initial study analyzed patients with late-stage disease, simulations of detection of circulating tumor DNA in earlier-stage disease indicate that the sensitivity of this approach is largely dependent on the amount of sequence data obtained. Currently, cell-free DNA analyses could be used to provide information about patient's tumor and guide clinical management. As the cost of sequencing continues to decrease, this approach and related methods [6,7] may provide a feasible avenue for profiling circulating DNA for early tumor detection.

**Figure 1:**
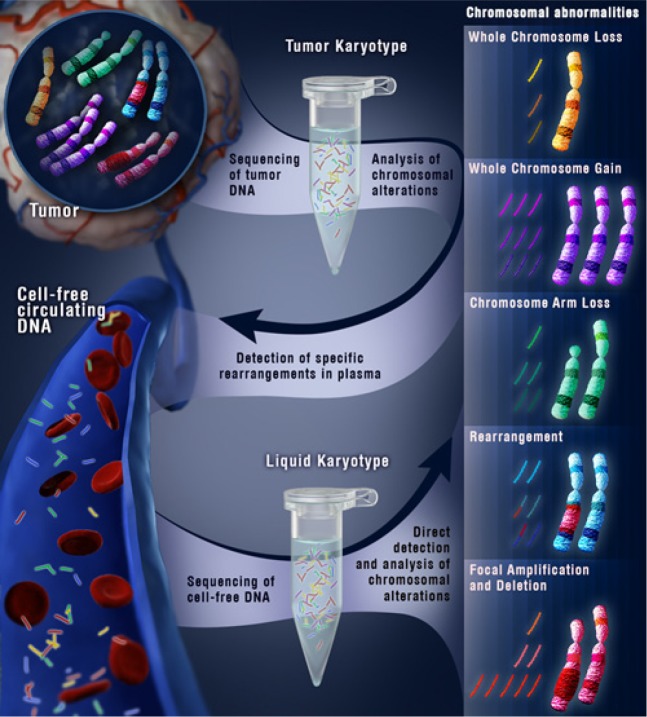
Schematic of cell-free genomic analyses to detect structural alterations in human cancer.
